# A Comparison of En Face Optical Coherence Tomography and Fundus Autofluorescence in Stargardt Disease

**DOI:** 10.1167/iovs.17-22532

**Published:** 2017-10

**Authors:** Vivienne C. Greenstein, Jason Nunez, Winston Lee, Kaspar Schuerch, Brad Fortune, Stephen H. Tsang, Rando Allikmets, Janet R. Sparrow, Donald C. Hood

**Affiliations:** 1 Department of Ophthalmology, Columbia University, New York, New York, United States; 2 Department of Psychology, Columbia University, New York, New York, United States; 3 Devers Eye Institute, Portland, Oregon, United States; 4 Department of Pathology & Cell Biology, Columbia University, New York, New York, United States

**Keywords:** wide-field en face optical coherence tomography, Stargardt disease, fundus autofluorescence

## Abstract

**Purpose:**

To compare morphologic changes on en face images derived from wide-field swept-source optical coherence tomography (ssOCT) to hypo- and hyperautofluorescent (hypoAF, hyperAF) areas on short-wavelength autofluorescence (SW-AF), and near-infrared (NIR)-AF in recessive Stargardt disease (STGD1).

**Methods:**

Wide-field ssOCT cube scans were obtained from 16 patients (16 eyes). Averaged B-scans and SW-AF images were obtained using Spectralis HRA+OCT. NIR-AF images were obtained from 6 eyes. The inner/outer segment (IS/OS), OS/RPE, and RPE/Bruch's membrane boundaries were segmented, and en face slab images generated. A subRPE slab image was used to measure the abnormal RPE area, and an IS/OS slab image, the IS/OS junction loss area. These were compared to hypo- and abnormal SW-AF areas, and hypoNIR-AF areas. A preRPE(OS) slab image was used to evaluate the spatial and intraretinal locations of flecks.

**Results:**

For all eyes, RPE atrophy was visualized as a central hyperreflective area on the subRPE slab, and IS/OS junction loss as an abnormal reflective area on the IS/OS slab; the latter was significantly larger (*P* = 0.04). There was good agreement between the hyperreflective area on the subRPE slab image and hypoSW-AF area, and between the abnormal reflective area on the IS/OS slab and hypo-hyperSW-AF area; the hypoNIR-AF area indicated that the hyperreflective area on the subRPE slab underestimated RPE atrophy. The spatial locations of hyperreflective flecks on the en face preRPE(OS) slab image corresponded to those on the SW-AF images.

**Conclusions:**

Wide-field en face OCT imaging has the potential to be a clinically useful tool for the management of STGD1.

Recessive Stargardt disease (STGD1), a form of juvenile macular degeneration, is caused by more than 1000 mutations in the photoreceptor-specific *ABCA4* gene, which encodes the ATP-binding cassette transporter in photoreceptors.^1,2^ The transporter facilitates the clearance of all-trans–retinaldehyde,^3^ which is generated by photon-mediated *cis-trans* isomerization of retinaldehyde in the photoreceptor cell. When the transport activity is reduced or absent, the result is accelerated accumulation of lipofuscin in RPE cells.^4^ The increase in bisretinoid lipofuscin can be visualized as elevated fundus autofluorescence.^5^ The accumulation of lipofuscin in RPE cells is toxic, and leads to RPE and photoreceptor cell degeneration.^6,7^ This clinically, and genetically, heterogeneous disease is characterized by progressive atrophy in the central macular area, accompanied by symptoms of progressive loss of central visual function during the first two decades of life.^8–10^ On fundus examination, the atrophy in the central macula often is accompanied by a distribution of yellowish white deposits or “flecks” visible at the posterior pole and sometimes in the mid-periphery.^11^ Initially the flecks appear to be well-defined, but with disease progression confluence increases across the posterior pole and they resorb leaving residual atrophy. In addition, their spatial distribution changes over time; typically they spread in a centrifugal direction from the fovea,^12^ but other patterns have been found in individual patients.^7^

Several noninvasive imaging modalities are used for the diagnosis and monitoring of disease progression in STGD1. These include short wavelength fundus autofluorescence (SW-AF), near infrared autofluorescence (NIR-AF),^13,14^ and spectral domain optical coherence tomography (SD-OCT). SW-AF imaging reveals areas of decreased autofluorescence or hypoautofluorescence (hypoSW-AF), and areas of hyperautofluorescence (hyperSW-AF) in the form of flecks and/or rings. With SD-OCT it has been shown that the area of abnormal SW-AF (total hypoSW-AF and hyperSW-AF) in the central macula is associated with loss of the ellipsoid zone band (aka inner/outer segment [IS/OS] junction) and loss of the RPE as revealed by NIR-AF imaging.^13–17^ In one study, a high correlation between photoreceptor loss and the extent of abnormal SW-AF was found.^15^

However, the exact sequence of retinal degeneration occurring with disease progression remains controversial. Depending on the imaging modality, it has been suggested that photoreceptor loss occurs before RPE loss,^18,19^ that RPE cell loss and/or dysfunction occurs and then there is secondary photoreceptor cell loss,^13,14^ and that changes in the photoreceptor layer may occur simultaneously with the development of abnormalities in the RPE layer.^20^ In addition, the intraretinal position of the hyperSW-AF flecks, which often are observed on SW-AF, also is of interest. Since the autofluorescent appearance of flecks usually is associated with lipofuscin in the RPE, it has been assumed that they are to be found at the level of the RPE.^20–22^ However, in NIR-AF images, flecks are dark due to loss of RPE, while in horizontal SD-OCT images they correspond to hyperreflective deposits that are anterior to RPE/Bruch's membrane and traverse photoreceptor-attributable OCT layers.^12,23^ There now is abundant evidence in human disease and mouse models that degenerating photoreceptor cells generate bisretinoid at elevated levels and it is reasonable to suggest that the SW-AF of flecks originates in degenerating photoreceptor cells.

We used an en face imaging approach to gain further insight into the morphologic changes that accompany macular atrophy, and corroborated our understanding of the intraretinal location of flecks in patients with STGD1. Advances in OCT technology, such as increased speed of acquisition of images, improved resolution, and improvements in software and data processing, have enabled rapid and powerful visualization of en face OCT projection images. The en face visualization approach provides a coronal view of a particular layer within the central retina by isolating its reflections from a specified depth. Thus, it aids in the precise localization and measurement of lesions within specific retinal layers. It also provides the ability to register the resulting en face images to other fundus imaging modalities, such as fundus autofluorescence (FAF), using retinal vessels and other landmarks (e.g., see prior studies^24,25^). In this study, we compared the morphologic changes visualized on en face images derived from wide-field swept-source (ss) OCT scans qualitatively and quantitatively to hypo- and hyperautofluorescent (hypoAF, hyperAF) areas detected with SW-AF.

## Methods

### Subjects

We studied 16 patients (16 eyes; 9 male and 7 female patients; mean age, 38.3 ± 17.7 years; range, 11–70 years), with at least one (expected) disease-causing mutation in the *ABCA4* gene. Demographic, genetic, and selected clinical data are presented in Table 1. All patients had a complete ocular examination, including best-corrected visual acuity with subjective refraction. Eyes were excluded from the study if there was evidence of significant ocular media opacities, refractive errors greater than ±6 diopter (D) sphere or ±2 D cylinder, elevated IOP > 21 mm Hg, and a history or diagnosis of any other significant ocular comorbidities. The eye with the higher image quality value for the wide-field OCT cube scan was included in the study. Best corrected visual acuity in the tested eye ranged from 0.01 to 1.3 logMAR (20/25–20/400, Snellen acuity).

**Table 1:**
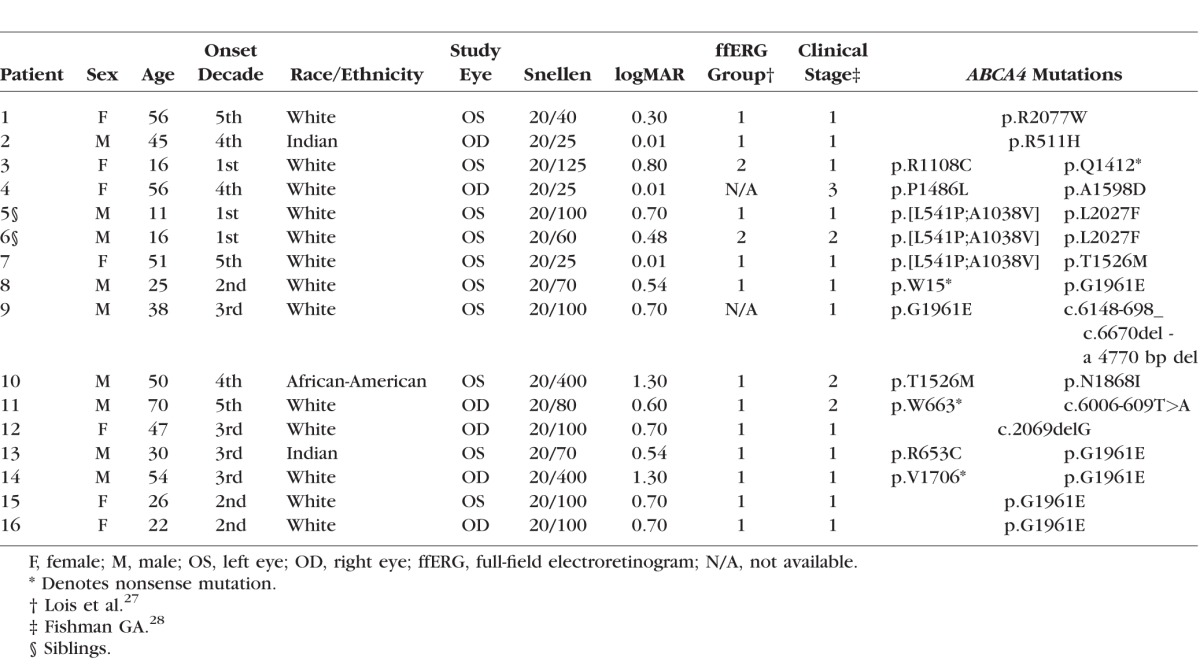
Clinical, Demographic, and Genetic Characteristics of Study Patients With Recessive Stargardt Disease

Full-field scotopic and photopic electroretinograms (ERGs) were obtained from both eyes of 14 patients according to the International Society for Clinical Electrophysiology of Vision (ISCEV) standards.^26^ Based on the full-field ERG results, patients were classified into group 1 if the scotopic and photopic ERG amplitudes were within normal limits (12 patients, 12 eyes), group 2 if the photopic ERG amplitudes were decreased (patients P3 and P6), or group 3 if the scotopic and photopic ERG amplitudes were decreased.^27^ In addition the disease was classified into one of four clinical stages or phenotypes based on those described by Fishman.^28^ A total of 12 patients had stage 1 (characterized by pigmentary changes in the macula ranging from nonspecific pigment mottling to the presence of small atrophic-appearing foveal lesions with localized parafoveal or perifoveal flecks), three stage 2 (presence of numerous yellowish-white flecks throughout the posterior pole) and one stage 3 (extensive macular atrophy with diffuse flecks throughout the fundus) disease (Table 1).

The study was performed under protocol AAAI9906 with the approval of the institutional review board of Columbia University. All study procedures complied with the Health Insurance Portability and Accountability Act of 1996. All patients were enrolled in accordance with the tenets set out in the Declaration of Helsinki. Informed consent was obtained before enrollment.

### Genetic Analyses

All patients were screened for *ABCA4* variants by complete sequencing of all coding and intron/exon boundaries of the gene by either Sanger or next-generation sequencing (NGS) as described previously,^29,30^ or with the Illumina TruSeq Custom Amplicon protocol (Illumina, San Diego, CA, USA), followed by sequencing on the Illumina MiSeq platform. The NGS reads were analyzed and compared to the reference genome GRCh37/hg19, using the variant discovery software NextGENe (SoftGenetics LLC, State College, PA, USA). All detected possibly disease-associated variants were confirmed by Sanger sequencing and analyzed with Alamut software (available in the public domain at http://www.interactive-biosoftware.com). Segregation of the variants with the disease was analyzed if family members were available. The allele frequencies of all variants were compared to the Exome Variant Server (EVS) dataset, NHLBI Exome Sequencing Project, Seattle, WA, USA (available in the public domain at http://snp.gs.washington.edu/EVS/; accessed March 2014). Genetic data are presented in Table 1. Two expected disease-causing *ABCA4* variants were detected in 11 patients and one mutation in the remaining five patients. Patient genotypes consisted mostly of known missense variants including the c.5882G>A (p.G1961E), harbored by six patients (P8, P9, P13, P14, P15, and P16), in addition to four stop-gain mutations and two small deletions, c.2069delG and c.6148-698_ c.6670del - a 4770 base pair (bp) deletion.

### Imaging

Following pupil dilation, SW-AF (488 nm excitation) 30° and 55° images were acquired with the Spectralis HRA+OCT (Heidelberg Engineering, Heidelberg, Germany) after a 20-second bleach of the photopigments in AF mode.^31^ Care was taken to obtain high-quality images with maximum field uniformity. In addition, a 9-mm high-resolution horizontal line scan through the fovea was obtained with the Spectralis. The SD-OCT scans were registered automatically to a simultaneously acquired near-infrared reflectance (NIR-R) fundus image. In addition NIR-AF (787 nm excitation) images were acquired from 6 of the 16 eyes with the Heidelberg Retina Angiograph 2 scanning laser ophthalmoscope (HRA2-cSLO, Heidelberg Engineering) using the indocyanine-green angiography mode (without injection of dye) after focus adjustment in infrared reflectance mode.

**Figure 1:**
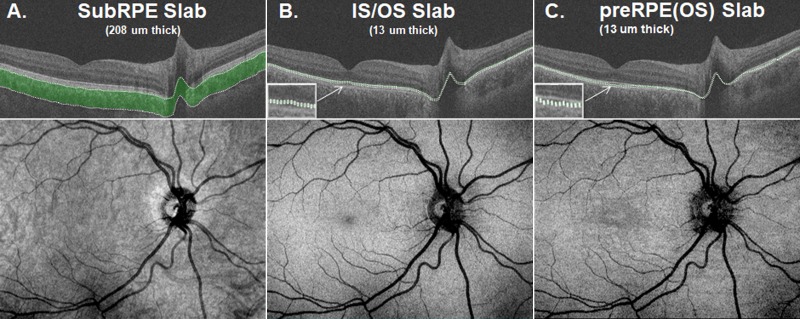
(A) SubRPE slab with a thickness of 208 μm. (B) IS/OS slab with a thickness of 13 μm. (C) preRPE(OS) slab with a thickness of 13 μm. The corresponding en face images for a healthy control are shown below.

Wide-field (12 × 9 mm) cube scans consisting of 256 B-scans, each with 512 A-scans, were obtained from all 16 eyes with ssOCT (DRI-OCT; Topcon, Inc., Tokyo, Japan). Following manual correction of the automated segmentation of the IS/OS junction, OS/RPE, and RPE/BM boundaries,^32^ special purpose software (ATL 3D-Suite; Fortune B, et al. *IOVS* 2014;55:ARVO E-abstract 2644) was used to generate en face slab images based on the average reflectance intensity for slabs of constant thickness.^33^ To obtain optimal visualization of the morphologic changes, we chose the following en face slabs: A subRPE slab with a thickness of 208 μm below the RPE/BM boundary; an IS/OS slab positioned on the IS/OS junction with a thickness of 13 μm; and a preRPE(OS) slab positioned just above the proximal RPE border and protruding into the IS/OS region with a thickness ranging from 13 μm. Examples of the three slabs together with their en face images for a healthy control are shown in Figures 1A to 1C. The thicknesses of the slabs were chosen based on a pilot study and on those used by Sodi et al.^18^ The subRPE slab image was used to measure the central RPE lesion (i.e., on the subRPE slab image the atrophic RPE area was visualized indirectly as a “hyperreflective area”; the increased OCT signal resulted from the loss of the RPE and choriocapillaris); the IS/OS slab image was used to measure the area of IS/OS junction loss; and the preRPE(OS) slab image was used to evaluate the spatial and intraretinal location of flecks. The areas of RPE atrophy and IS/OS junction loss were measured in mm^2^ by two independent observers using ImageJ64 (National Institutes of Health [NIH], Bethesda, MD, USA; available in the public domain at imagej.nih.gov/ij/). The quality of some of the en face IS/OS slab images affected the clarity of the boundaries of the area of IS/OS junction loss. For these cases, the locations chosen to mark the boundary were checked by comparing them to the locations of IS/OS junction loss on the corresponding individual B-scans. The areas of RPE atrophy and IS/OS junction loss then were compared respectively to the area of hypoAF (i.e., absent AF on the SW-AF image and central darkened area on the NIR-AF image) and to the area of abnormal AF (i.e., the hypo- and surrounding hyperAF areas on the SW-AF image). The latter also were measured using ImageJ64. Lastly the spatial and intraretinal locations of flecks observed on the preRPE(OS) slab image were compared to those on the SW-AF image. All en face, SW-AF, and NIR-AF images were aligned to each other using i2kRetina software (DualAlign LLC, Clifton Park, NY, USA) and Adobe Photoshop CS5 (Adobe, Mountain View, CA, USA).

### Statistical Analyses

Statistical analysis was performed using PRISM 5 (GraphPad Software, Inc., La Jolla, CA, USA). Bland-Altman plots were generated to assess the agreement between the en face and SW-AF measurements. The statistical significance of the differences between the subRPE and IS/OS areas, and between the hypoAF and total abnormal AF areas was tested with paired-samples *t*-tests using SPSS Statistics 20.0 for Mac (SPSS IBM, Chicago, IL, USA). In addition, to assess the agreement between the measurements made by the two observers, interclass correlation coefficients (ICC) were calculated in SPSS.

## Results

### Changes on SW-AF

**Figure 2:**
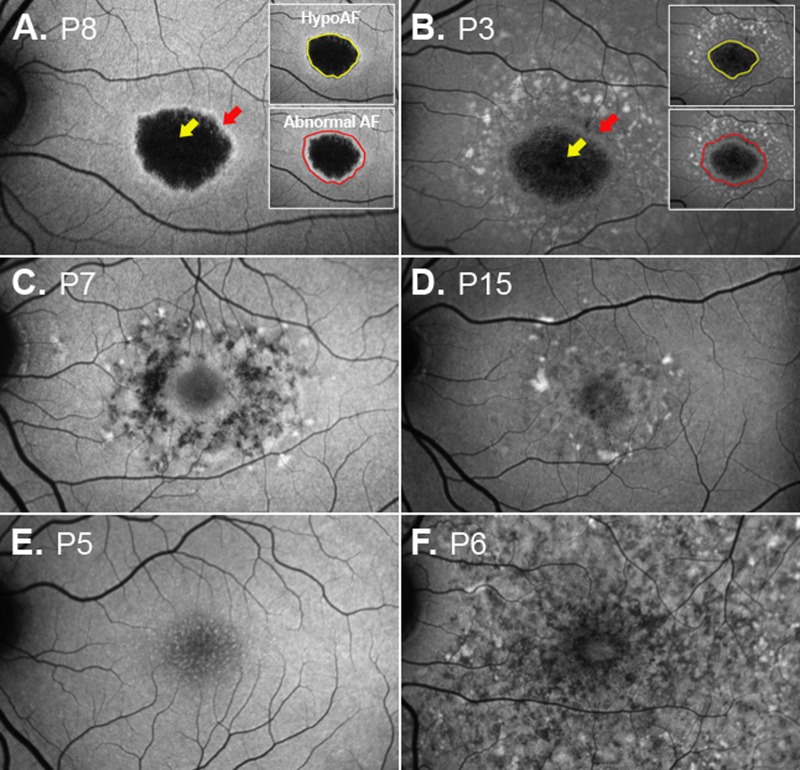
Examples of SW-AF images from six patients. (A) The central area of hypoAF (yellow arrow) is surrounded by a well-defined ring of hyperAF (red arrow). The central hypoAF area on the SW-AF image for P8 and the central area of abnormal AF are outlined in the insets. (B) The central area is surrounded by a mottled pattern of flecks (red arrow). (C, D) The fovea is encircled by hyper- and hypoAF flecks in the perifovea of varying size and shape. (E) Mottling on the central fovea. (F) Widespread hypo- and hyperAF changes.

**Figure 3:**
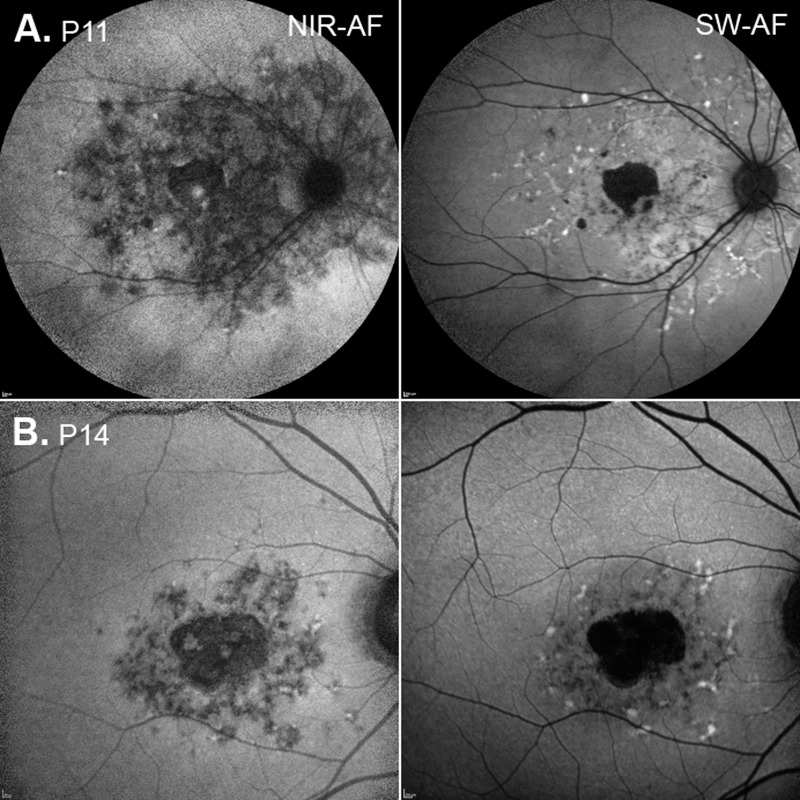
NIR and SW-AF images for P11 and P14.

All 16 eyes exhibited changes on SW-AF. The SW-AF images for 11 of the 16 eyes showed a central area of hypoAF of variable size (Figs. 2A, 2B, yellow arrows) surrounded by a well-defined border of hyperAF and/or a mottled pattern of flecks (Figs. 2A, 2B, red arrows). In three eyes, a pattern of hyper- and hypoAF flecks of varying size and shape was observed in the perifoveal region (Figs. 2B–D). Widespread hypo- and hyperAF changes extending beyond the arcades were observed in one eye (Fig. 2F), and mottling on the foveal center with no other visible areas of hypo- or hyperAF was observed in another eye (Fig. 2E). The areas of abnormal AF (i.e., the hypo- and surrounding hyperAF areas) and the areas of central hypoAF or absent AF were measured (see examples in Figs. 2A, 2B inserts). Unsurprisingly, for the 11 eyes with a central hypoAF area, the abnormal AF area was significantly larger (mean = 14.37 mm^2^, SD = 13.18 mm^2^) than the hypoAF area (mean = 4.66 mm^2^, SD = 4.57 mm^2^; *P* = 0.01; Table 2). For the six eyes with NIR-AF, the darkened areas in the NIR-AF images, indicating a reduction or absence of the NIR-AF signal, exhibited good correspondence to the areas of abnormal AF in the SW-AF images (see examples P11 and P14, Fig. 3).

**Table 2:**
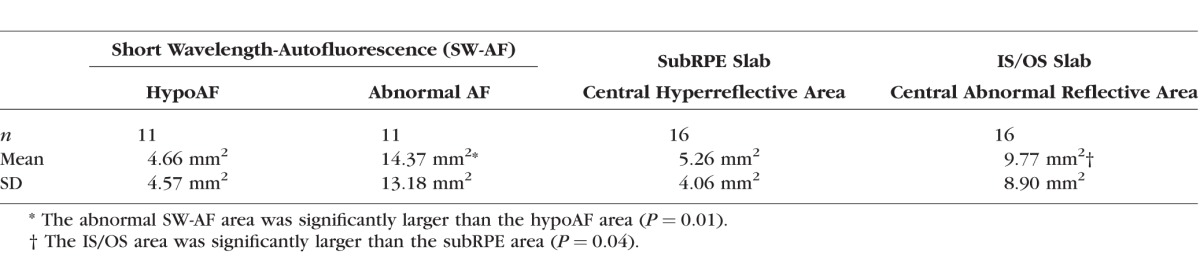
Summary of SW-AF and En Face OCT Measurements

### OCT En Face Slabs

**Figure 4:**
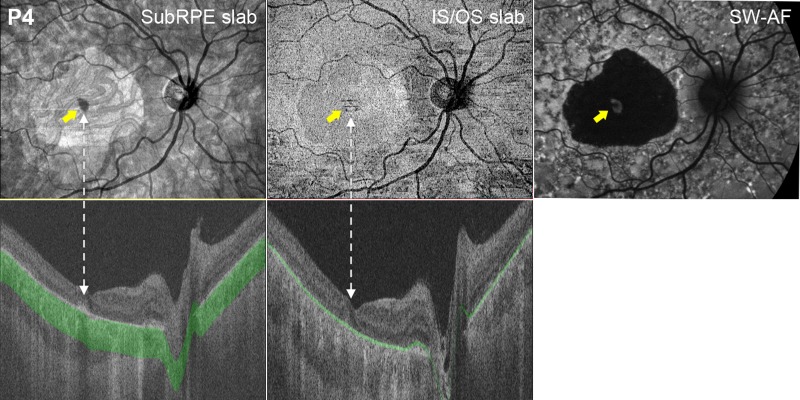
SubRPE, IS/OS en face, and SW-AF images for one patient, P4. The yellow arrows on the subRPE, IS/OS, and SW-AF images indicate foveal sparing. The subRPE slab image shows a central hyperreflective area. The corresponding en face IS/OS slab image shows a central relatively hyporeflective area surrounded by a hyperreflective border that appears to be larger than the area on the subRPE slab image. The SW-AF image shows a large central hypoAF area. The lower panels show horizontal ssOCT B-scans and respective subRPE slab and IS/OS slab.

**Figure 5:**
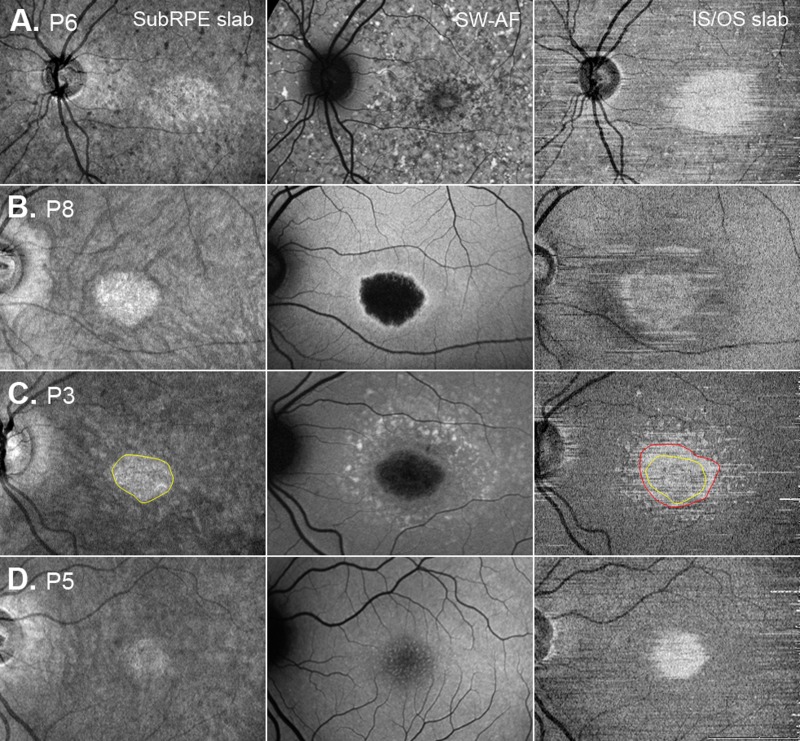
SubRPE, SW-AF, and IS/OS en face images for patients P6, P8, P3, and P5.

The subRPE slab images for the 11 eyes with central hypoAF on SW-AF showed a central hyperreflective area (see Figs. 4, 5A–D). The hyperreflectivity of this zone is attributable to scleral reflectance due to penetration of light into the choroid due to loss of the RPE and atrophy of the choriocapillaris^34^ as visualized in the ssOCT B-scan (Fig. 4, bottom left). As can be seen in Figures 4 and 5, the hyperreflective areas were similar in size and shape to the central hypoAF areas on SW-AF. For the subgroup of eyes with NIR-AF, these hyperreflective areas were smaller than the hypoNIR-AF areas.

Foveal sparing, present in four eyes (P1, P2, P4, and P7), also was an identifiable feature on the en face image. It was discernable as a hyporeflective area in the fovea, and as a relatively hyperAF area in the SW-AF image (Fig. 4, yellow arrows). Of interest, the subRPE slab image for the eye with widespread hypo- and hyperSW-AF changes (P6, Fig. 2F) showed a well-defined central hyperreflective area surrounded by hypo- and hyperreflective patterning (Fig. 5A), while the eye with foveal mottling on SW-AF (P5, Fig. 2E) exhibited an ill-defined central hyporeflective area on the subRPE slab image surrounded by an area of comparatively increased reflectivity (Fig. 5D).

The corresponding en face IS/OS slab images (Fig. 4, center column and Figs. 5A–D, right column) for these patients showed either a central hyperreflective area or a relatively hyporeflective area surrounded by a hyperreflective border. This central abnormal reflective area appeared to be larger than the area on the subRPE slab image (see Figs. 4 and 5A-D). To assess this quantitatively, these areas were measured on the IS/OS and subRPE slab images (see examples of areas in Fig. 5C). The en face IS/OS areas were larger than the subRPE areas. There was a significant difference between the measurements for the IS/OS area (mean = 9.77 mm^2^, SD = 8.90 mm^2^) and the subRPE area (mean = 5.26 mm^2^, SD = 4.06 mm^2^), *P* = 0.04 (see Table 2).

### Comparison of SW-AF and OCT En Face Slab Images

**Figure 6:**
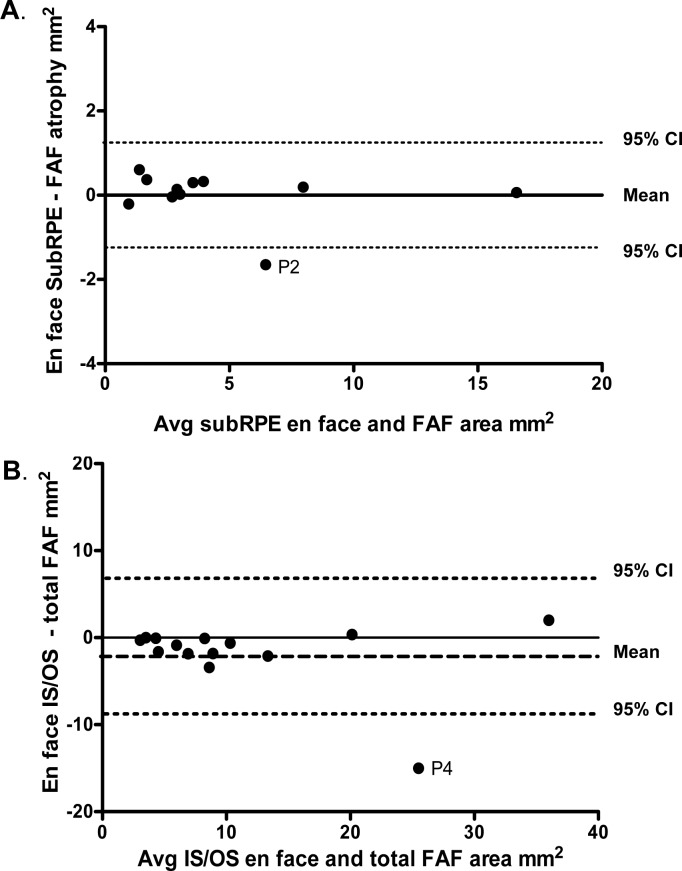
(A) Bland-Altman plot showing differences in measurements of the central hypoAF area on the SW-AF image to the central hyperreflective area on the subRPE en face image. (B) Bland-Altman plot showing differences in the size of the measurements of the abnormal AF area on the SW-AF image to the central hyporeflective area on the IS/OS en face image. The dashed horizontal lines represent 95% confidence intervals (CIs) and the interrupted line the mean.

To assess the agreement between the two techniques used to quantify the extent of RPE atrophy and IS/OS junction loss, measurements of the central hypoAF area on the SW-AF image were compared to those of the central hyperreflective area on the subRPE en face image, and measurements of the abnormal AF area on the SW-AF image were compared to the central abnormal reflective (central hyperreflective or hyporeflective area and surrounding hyperreflective border) area on the IS/OS en face image. The Bland-Altman plot in Figure 6A illustrates the similarity of these measurements for the two techniques. There was no evidence of bias regarding the comparison between measurements of the central hypoAF area on SW-AF and the subRPE en face measurements. The Bland-Altman plot in Figure 6B comparing the abnormal AF area on the SW-AF image to the central abnormal reflective area on the IS/OS en face image also showed similarity of measurements for the two techniques; however on average, the SW-AF measurements of the abnormal AF area were slightly larger than the en face IS/OS measurements (1.8 mm^2^). Average ICCs revealed excellent agreement between the two independent observers for all area measurements (range, 0.93–0.98).

### Retinal Flecks

**Figure 7:**
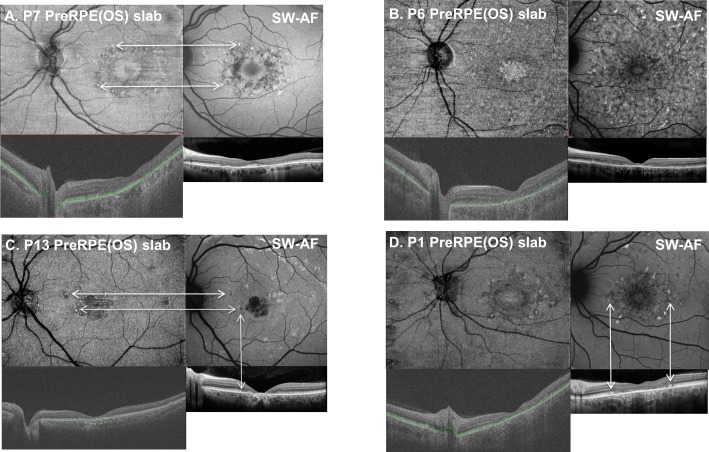
Examples of the en face preRPE(OS) slab images with corresponding SW-AF, foveal ssOCT, and SD-OCT scan images for four patients. The horizontal arrows in (A) and (C) indicate that the spatial locations of the hyperreflective flecks correspond to those on the SW-AF images. The vertical arrows in (C) and (D) connecting the SW-AF images to the horizontal foveal SD-OCT scan indicate the intraretinal position of the flecks.

Retinal flecks of varying shape and size were identified on the SW-AF images of 11 of the 16 eyes. The hyper- and hypoAF flecks were located in the perifoveal region and/or diffusely throughout the fundus (Figs. 2B–D, 2F). The preRPE(OS) slab provided the best means for detecting flecks. On the en face preRPE(OS) image (Figs. 7A–D, upper left), they were apparent as small hyperreflective structures either surrounding the central hyporeflective area or widely distributed throughout the image; a hyporeflective border surrounded some of the flecks. The spatial locations of the hyperreflective flecks corresponded to those on the SW-AF images (Figs. 7A, 7C). The ssOCT and SD-OCT scans provided information regarding the intraretinal location of the flecks. For example, on the SD-OCT B-scans through the fovea (Figs. 7A–D, lower right), the flecks appear as hyperreflective regions that project anterior to RPE/Bruch's membrane disrupting the IS/OS junction band. In some cases they extend toward the outer limiting membrane (Figs. 7C, 7D, lower right).

## Discussion

The morphologic changes on en face images derived from wide-field ssOCT scans were compared qualitatively and quantitatively to changes visible on SW-AF. In agreement with previous reports,^18,35^ a central hyperreflective area attributable to RPE atrophy was observed on the en face subRPE slab image (Figs. 4, 5). The area was similar in size and shape to the hypoAF area in the central macula in the SW-AF image. Consistent with observations by Sodi et al.,^18^ measurements of the hyperreflective area on the en face subRPE slab image (i.e., the area of RPE atrophy) revealed that this lesion area was significantly smaller than the area of abnormal reflectance on the IS/OS slab image (i.e., the area related to IS/OS junction loss). However, it also was smaller than the area of RPE cell loss identified by NIR-AF.

Our findings provided some insight into the possible sequence of outer retinal degenerative changes in STGD1. First, we assume that IS/OS junction loss is correlated with photoreceptor loss and/or dysfunction;^15,17^ that hypo–SW-AF or absent SW-AF in STGD1 represents RPE loss and/or atrophy; that hyper–SW-AF can be attributed to accelerated bisretinoid production from degenerating photoreceptor cells; and that it is associated with RPE cells being absent or with thinning and dysfunctional RPE.^13,36^ Second, our SW-AF results indicated that the area of abnormal AF was significantly larger than the hypo- or absent AF area, and that it included the area of IS/OS junction loss. However, these observations cannot be interpreted as evidence that photoreceptor degeneration precedes RPE atrophy in STGD1. Additionally, the tested eye of P5 at the initial visit showed IS/OS junction loss in the fovea on ssOCT and SD-OCT, and central mottling in the SW-AF image that would be indicative of RPE atrophy (see Figs. 2D, 5D). This interpretation raises questions about the results of previous studies comparing NIR- and SW-AF in patients with STGD1. In these studies, the zone of decreased NIR-AF was reported to be larger than the zone of reduced or abnormal SW-AF and more closely related to the extent of the IS/OS junction loss seen on SD-OCT.^13,14^ As the main source of NIR-AF is melanin in the RPE and choroid, it was suggested that the results were consistent with RPE cell loss preceding photoreceptor cell degeneration.^13,14^ In the current study, NIR-AF images were obtained from six of the 16 patients and in agreement with previous studies the central area of absent or hypoAF was larger on NIR than SW-AF.^13,14^ In addition, the lesion area, indicated by darkening in the NIR-AF image, was larger than the en face subRPE area. When the surrounding clusters of hypoAF, that is, patchy hypoAF areas on NIR-AF, were included in the measurement, the total area was greater than either the en face IS/OS or abnormal SW-AF areas (see Fig. 3). Since the NIR-AF signal is produced, for the most part, from RPE melanin, absence of NIR-AF is indicative of loss of RPE cells. Residual levels of NIR-AF could be accounted for readily by melanin-containing RPE debris from the degenerating cells. If the latter is assumed, the combined NIR-, SW-AF and en face results suggested that photoreceptor cell degeneration has occurred in the presence of dead and/or dying dysfunctional RPE cells.

We also were interested in determining whether the wide-field en face technique could be used to corroborate our previous findings related to the intraretinal location of flecks in patients with STGD1.^7^ These retinal flecks are an important clinical feature of STGD1. We found that the en face preRPE(OS) slab image provided the best means for detecting flecks. They were evident as small hyperreflective structures either surrounding the central hyporeflective area or widely distributed throughout the image. A hyporeflective border surrounded some of the structures. They corresponded spatially to hyperAF flecks identified on SW-AF images (see examples, Figs. 7A, 7C, 7D) and to darkened foci in NIR-AF images as has been described previously.^7^ With regard to their intraretinal location, it had been assumed that the AF of flecks in SW-AF images originated from RPE cells since SW-AF is associated with lipofuscin in the RPE.^20–22^ However, horizontal ss- and SD-OCT B-scans through the fovea (Figs. 7A–D, lower), revealed that, in some cases, the flecks extended anteriorly through photoreceptor attributable bands and interrupted the IS/OS junction band, and even the band attributable to the outer limiting membrane (Fig. 7D). These cases are in agreement with those of Sparrow et al.,^7^ who reported that flecks identified in SW-AF images corresponded to hyperreflective deposits traversing photoreceptor-attributable bands in SD-OCT scans. The latter lesions are interpreted as being indicative of degenerating photoreceptor cells.

Our results using the wide-field en face approach have implications for the clinician. They suggested that en face wide-field ssOCT has the potential to be a clinically useful tool in the management of patients with STGD1. In agreement with recent studies, we have shown that the subRPE slab enhances visualization of RPE atrophy.^18,35^ We also have shown that the IS/OS slab provides the best means for visualizing IS/OS junction loss, and the preRPE(OS) slab for flecks. In addition, when we compared the en face sub-RPE measurements to the central hypoAF area measurements on SW-AF, and the en face IS/OS measurements to the abnormal AF measurements, we found good agreement. The results of the Bland-Altman plots suggested that the en face wide-field ssOCT technique can be used as a complementary test to SW-AF, the current clinical gold standard, for measuring and monitoring changes in RPE atrophy. Moreover, the en face approach we used has the added advantage that the clinician can visualize and measure the area of IS/OS junction loss, a marker of photoreceptor loss, inspect the cross-sectional scans to obtain information regarding the status of the RPE and photoreceptor layers, and determine the intraretinal location of flecks. The use of the wide-field ssOCT technique can serve as a complimentary test to SW-AF for detecting and monitoring changes at various stages of the disease whether SW-AF changes present as moderately defined mottling as in Figure 2E, or are widespread as in Figure 2F. In both cases, we were able to observe, and measure, a central hyperreflective area indicating RPE atrophy on the en face subRPE slab image.

One of the limitations of the en face approach is that it is dependent on reliable segmentation of the IS/OS junction band and RPE/BM boundary. We found that we had to correct some of the automated segmentation of these boundaries manually, particularly in the region of the lesion. In addition, the quality of some of the en face IS/OS slab images affected the clarity of the boundaries of IS/OS junction loss. For these cases, we checked the accuracy of our en face IS/OS area measurements by comparing them to the extent of IS/OS junction loss on the corresponding individual B-scans. Another limitation of the current study was that comparison with NIR-AF imaging was possible only in a small subgroup of patients. Despite these limitations and the small sample size used in this study, we demonstrated that the wide-field en face slab approach has the potential to be a useful clinical tool for detecting and monitoring changes in RPE atrophy, IS/OS junction loss, and fleck distribution in patients with STGD1. In addition, a comparison between en face and SW-AF results has contributed to our understanding of the spatial and intraretinal location of flecks, a prominent clinical feature of STGD1.

## References

[1] Allikmets R, Singh N, Sun H, A photoreceptor cell- specific ATP-binding transporter gene (ABCR) is mutated in recessive Stargardt macular dystrophy. *Nat Genet*. 1997; 15: 236– 246. 9054934 10.1038/ng0397-236

[2] Cornelis SS, Bax NM, Zernant J, In silico functional meta-analysis of 5,962 ABCA4 variants in 3,928 retinal dystrophy cases. *Hum Mutat*. 2017; 38: 400– 408. 28044389 10.1002/humu.23165

[3] Quazi F, Molday RS. ATP-binding cassette transporter ABCA4 and chemical isomerization protect photoreceptor cells from the toxic accumulation of excess 11-cis-retinal. *Proc Natl Acad Sci U S A*. 2014; 111: 5024– 5029. 24707049 10.1073/pnas.1400780111 PMC3977269

[4] Weng J, Mata NL, Azarian SM, Tzekov RT, Birch DG, Travis GH. Insights into the function of Rim protein in photoreceptors and etiology of Stargardt's disease from the phenotype in abcr knockout mice. *Cell*. 1999; 98: 13– 23. 10412977 10.1016/S0092-8674(00)80602-9

[5] Burke TR, Duncker T, Woods RL, Quantitative fundus autofluorescence in recessive Stargardt disease. *Invest Ophthalmol Vis Sci*. 2014; 55: 2841– 2852. 24677105 10.1167/iovs.13-13624 PMC4008047

[6] Cideciyan AV, Aleman TS, Swider M, Mutations in ABCA4 result in accumulation of lipofuscin before slowing of the retinoid cycle: a reappraisal of the human disease sequence. *Hum Mol Genet*. 2004; 13: 525– 534. 14709597 10.1093/hmg/ddh048

[7] Sparrow JR, Marsiglia M, Allikmets R, Flecks in recessive Stargardt disease: short-wavelength autofluorescence, near-infrared autofluorescence, and optical coherence tomography. *Invest Ophthalmol Vis Sci*. 2015; 56: 5029– 5039. 26230768 10.1167/iovs.15-16763 PMC4525681

[8] Rotenstreich Y, Fishman GA, Anderson RJ. Visual acuity loss and clinical observations in a large series of patients with Stargardt disease. *Ophthalmology*. 2003; 110: 1151– 1158. 12799240 10.1016/S0161-6420(03)00333-6

[9] Armstrong JD, Meyer D, Xu S, Elfervig JL. Long-term follow-up of Stargardt's disease and fundus flavimaculatus. *Ophthalmology*. 1998; 105: 448– 457. 9499775 10.1016/S0161-6420(98)93026-3

[10] Fishman GA, Farber M, Patel BS, Derlacki DJ. Visual acuity loss in patients with Stargardt's macular dystrophy. *Ophthalmology*. 1987; 94: 809– 814. 3658351 10.1016/s0161-6420(87)33533-x

[11] Walia S, Fishman GA, Kapu R. Flecked-retinal syndromes. *Ophthalmic Genet*. 2009; 30: 69– 75. 19373677 10.1080/13816810802654516

[12] Cukras CA, Wong WT, Caruso R, Cunningham D, Zein W, Sieving PA. Centrifugal expansion of fundus autofluorescence patterns in Stargardt disease over time. *Arch Ophthalmol*. 2012; 130: 171– 179. 21987580 10.1001/archophthalmol.2011.332 PMC3768260

[13] Duncker T, Marsiglia M, Lee W, Correlations among near- infrared and short-wavelength autofluorescence and spectral domain optical coherence tomography in recessive Stargardt disease. *Invest Ophthalmol Vis Sci*. 2014; 55: 8134– 8143. 25342616 10.1167/iovs.14-14848 PMC4266077

[14] Greenstein VC, Schuman AD, Lee W, Near-infrared autofluorescence: its relationship to short-wavelength autofluorescence and optical coherence tomography in recessive Stargardt disease. *Invest Ophthalmol Vis Sci*. 2015; 56: 3226– 3234. 26024107 10.1167/iovs.14-16050 PMC4453463

[15] Ergun E, Hermann B, Wirtitsch M, Assessment of central visual function in Stargardt's disease/fundus flavimaculatus with ultrahigh-resolution optical coherence tomography. *Invest Ophthalmol Vis Sci*. 2005; 46: 310– 316. 15623790 10.1167/iovs.04-0212

[16] Srinivasan VJ, Wojtkowski M, Witkin AJ, High-definition and 3-dimensional imaging of macular pathologies with high- speed ultrahigh-resolution optical coherence tomography. *Ophthalmology*. 2006; 113: 2054 e1– e14. 10.1016/j.ophtha.2006.05.046 PMC1939823 17074565

[17] Burke TR, Rhee DW, Smith RT, Quantification of peripapillary sparing and macular involvement in Stargardt disease (STGD1). *Invest Ophthalmol Vis Sci*. 2011; 52: 8006– 8015. 21873672 10.1167/iovs.11-7693 PMC3220414

[18] Sodi A, Mucciolo DP, Cipollini F, En face OCT in Stargardt disease. *Graefes Arch Clin Exp Ophthalmol*. 2016; 254: 1669– 1679. 26743751 10.1007/s00417-015-3254-1

[19] Gomes NL, Greenstein VC, Carlson JN, A comparison of fundus autofluorescence and retinal structure in patients with Stargardt disease. *Invest Ophthalmol Vis Sci*. 2009; 50: 3953– 3959. 19324865 10.1167/iovs.08-2657 PMC2749553

[20] Ritter M, Zotter S, Schmidt WM, Characterization of Stargardt disease using polarization-sensitive optical coherence tomography and fundus autofluorescence imaging. *Invest Ophthalmol Vis Sci*. 2013; 54: 6416– 6425. 23882696 10.1167/iovs.12-11550

[21] Fujinami K, Sergouniotis PI, Davidson AE, Clinical and molecular analysis of Stargardt disease with preserved foveal structure and function. *Am J Ophthalmol*. 2013; 156: 487– 501. 23953153 10.1016/j.ajo.2013.05.003

[22] Sisk RA, Leng T. Multimodal imaging and multifocal electroretinography demonstrate autosomal recessive Stargardt disease may present like occult macular dystrophy. *Retina*. 2014; 34: 1567– 1575. 24743636 10.1097/IAE.0000000000000136

[23] Voigt M, Querques G, Atmani K, Analysis of retinal flecks in fundus flavimaculatus using high-definition spectral-domain optical coherence tomography. *Am J Ophthalmol*. 2010; 150: 330– 337. 20579629 10.1016/j.ajo.2010.04.001

[24] Podoleanu AG, Dobre GM, Webb DJ, Jackson DA. Simultaneous en-face imaging of two layers in the human retina by low-coherence reflectometry. *Optics Letters*. 1997; 22: 1039– 1041. 18185745 10.1364/ol.22.001039

[25] Srinivasan VJ, Adler DC, Chen Y, Ultrahigh-speed optical coherence tomography for three-dimensional and en face imaging of the retina and optic nerve head. *Invest Ophthalmol Vis Sci*. 2008; 49: 5103– 5110. 18658089 10.1167/iovs.08-2127 PMC2743183

[26] McCulloch DL, Marmor MF, Brigell MG, ISCEV Standard for full-field clinical electroretinography (2015 update). *Doc Ophthalmol*. 2015; 130: 1– 12. 10.1007/s10633-014-9473-7 25502644

[27] Lois N, Holder GE, Bunce C, Fitzke FW, Bird AC. Phenotypic subtypes of Stargardt macular dystrophy-fundus flavimaculatus. *Arch Ophthalmol*. 2001; 119: 359– 369. 11231769 10.1001/archopht.119.3.359

[28] Fishman GA. Fundus flavimaculatus. A clinical classification. *Arch Ophthalmol*. 1976; 94: 2061– 2067. 999551 10.1001/archopht.1976.03910040721003

[29] Jaakson K, Zernant J, Kulm M, Genotyping microarray (gene chip) for the ABCR (ABCA4) gene. *Hum Mutat*. 2003; 22: 395– 403. 14517951 10.1002/humu.10263

[30] Zernant J, Schubert C, Im KM, Analysis of the ABCA4 gene by next-generation sequencing. *Invest Ophthalmol Vis Sci*. 2011; 52: 8479– 8487. 21911583 10.1167/iovs.11-8182 PMC3208189

[31] Delori F, Greenberg JP, Woods RL, Quantitative measurements of autofluorescence with the scanning laser ophthalmoscope. *Invest Ophthalmol Vis Sci*. 2011; 52: 9379– 9390. 22016060 10.1167/iovs.11-8319 PMC3250263

[32] Yang Q, Reisman CA, Chan K, Ramachandran R, Raza A, Hood DC. Automated segmentation of outer retinal layers in macular OCT images of patients with retinitis pigmentosa. *Biomed Opt Express*. 2011; 2: 2493– 2503. 21991543 10.1364/BOE.2.002493 PMC3184859

[33] Hood DC, Fortune B, Mavrommatis MA, Details of glaucomatous damage are better seen on OCT en face images than on OCT retinal nerve fiber layer thickness maps. *Invest Ophthalmol Vis Sci*. 2015; 56: 6208– 6216. 26426403 10.1167/iovs.15-17259 PMC4703406

[34] Yehoshua Z, Garcia Filho CA, Penha FM, Comparison of geographic atrophy measurements from the OCT fundus image and the sub-RPE slab image. *Ophthalmic Surg Lasers Imaging Retina*. 2013; 44: 127– 132. 23510038 10.3928/23258160-20130313-05

[35] Melillo P, Testa F, Rossi S, En face spectral-domain optical coherence tomography for the monitoring of lesion area progression in Stargardt disease. *Invest Ophthalmol Vis Sci*. 2016; 57: 247– 252. 10.1167/iovs.15-18751 PMC4968920 27409479

[36] Gelman R, Chen R, Blonska A, Barile G, Sparrow JR. Fundus autofluorescence imaging in a patient with rapidly developing scotoma. *Retinal Cases Brief Rep*. 2012; 6: 345– 348. 10.1097/ICB.0b013e318260af5d PMC3535223 23293707

